# Direct Detection of *Orthoflavivirus* via Gold Nanorod Plasmon Resonance

**DOI:** 10.3390/s25154775

**Published:** 2025-08-03

**Authors:** Erica Milena de Castro Ribeiro, Bruna de Paula Dias, Cyntia Silva Ferreira, Samara Mayra Soares Alves dos Santos, Rajiv Gandhi Gopalsamy, Estefânia Mara do Nascimento Martins, Cintia Lopes de Brito Magalhães, Flavio Guimarães da Fonseca, Luiz Felipe Leomil Coelho, Cristiano Fantini, Luiz Orlando Ladeira, Lysandro Pinto Borges, Breno de Mello Silva

**Affiliations:** 1 Department of Biological Sciences, Federal University of Ouro Preto, Ouro Preto 35402-136, Brazil; emcribeiro@gmail.com (E.M.d.C.R.); bdiascmt@gmail.com (B.d.P.D.); csf.ferreira@gmail.com (C.S.F.); samara.santos@aluno.ufop.edu.br (S.M.S.A.d.S.); cintia.magalhaes@ufop.edu.br (C.L.d.B.M.); 2 Oswaldo Cruz Foundation, René Rachou Institute, FIOCRUZ, Belo Horizonte 30190-009, Brazil; 3 Division of Phytochemistry and Drug Design, Department of Biosciences, Rajagiri College of Social Sciences (Autonomous), Kochi 683104, Kerala, India; egarajiv@gmail.com; 4 Nuclear Technology Development Center, CDTN, Belo Horizonte 31270-901, Brazil; estefaniamartinsnanobio@gmail.com; 5 Department of Microbiology, Institute of Biological Sciences, Federal University of Minas Gerais, Belo Horizonte 31270-901, Brazil; fdafonseca@icb.ufmg.br; 6 Vaccine Laboratory, Department of Microbiology and Immunology, Federal University of Alfenas, Alfenas 37130-001, Brazil; luiz.coelho@unifal-mg.edu.br; 7 Department of Physics, Federal University of Minas Gerais, Belo Horizonte 31270-901, Brazil; fantini.cris@gmail.com (C.F.); loladeira@gmail.com (L.O.L.); 8 Department of Pharmacy, Federal University of Sergipe, São Cristóvão 49400-000, Brazil

**Keywords:** flavivirus, gold nanorods, localized surface plasmon resonance, diagnostic, nanotechnology

## Abstract

Dengue, Zika, yellow fever, chikungunya, and Mayaro arboviruses represent an increasing threat to public health because of the serious infections they cause annually in many countries. Serological diagnosis of these viruses is challenging, making the development of new diagnostic strategies imperative. In this study, we investigated the effectiveness of gold nanorods (GNRs) functionalized with specific anti-dengue and anti-orthoflavivirus antibodies in detecting viral particles. GNRs were created with a length-to-width ratio of up to 5.5, a size of 71.4 ± 6.5 nm, and a light absorption peak at 927 nm, and they were treated with 4 mM polyethyleneimine. These GNRs were attached to a small amount of monoclonal antibodies that target flaviviruses, and the viral particles were detected by measuring the localized surface plasmon resonance using an UV-Vis/NIR spectrometer. The tests found *Orthoflavivirus dengue* and *Orthoflavivirus zikaense* in diluted human serum and ground-up mosquitoes, with the lowest detectable amount being 100 PFU/mL. The GNRs described in this study can be used to enhance flavivirus diagnostic tests or to develop new, faster, and more accurate diagnostic techniques. Additionally, the functionalized GNRs presented here are promising for supporting virological surveillance studies in mosquitoes. Our findings highlight a fast and highly sensitive method for detecting *Orthoflavivirus* in both human and mosquito samples, with a detection limit as low as 100 PFU/mL.

## 1. Introduction

*Orthoflaviviruses* (renamed *Flavivirus* in 2023) include several enveloped viruses with 11-kilobase single-stranded positive-sense RNA genomes transmitted to humans by arthropod vectors [1]. Some of these viruses, like Dengue (DENV), Zika (ZIKV), Japanese encephalitis (JEV), West Nile (WNV), and Yellow fever (YFV), are known to cause serious illnesses that affect health worldwide [2]. In recent years, these viruses have spread throughout the tropics and become endemic in more than 100 countries in the subtropics, coinciding with the distribution of the mosquito vector *Aedes aegypti* [2,3]. Therefore, epidemiological control that facilitates the prevention of new outbreaks and the implementation of early action control plans is increasingly necessary, especially in identifying viral introduction and spread by travelers and vectors [4].

The clinical diagnosis of infections caused by *Orthoflaviviruses* is usually based on nonspecific symptom analysis, which makes accurate and timely detection difficult [5,6]. Molecular and serological tests have confirmed viral infections in clinical samples [7,8]. PCR, considered the gold standard, has high specificity, but its application is limited by cost, technical requirements, and infrastructure demands. On the other hand, serological tests are faster and more accessible; however, they present a risk of cross-reactivity, especially in regions with co-circulation of *Alphavirus chikungunya*. In addition, these tests have limited sensitivity, leading to false-negative results [9,10].

Given the absence of licensed vaccines for most *Orthoflaviviruses*, the lack of effective antiviral drugs, and the increasing resistance of vectors to insecticides, monitoring viruses in field-collected mosquito vectors is essential to assess the risks associated with *Orthoflaviviruses* and implement effective control strategies [11,12,13,14,15].

Detection of arboviruses in mosquitoes involves methods such as viral isolation and culture, ELISA, or viral RNA extraction with RT-PCR confirmation; however, these approaches also share limitations related to cost, time, and infrastructure [11,16]. Thus, there is a need to develop more practical, effective, accessible, and rapid *Orthoflavivirus* detection methods [12] for both mosquito surveillance and diagnosis in patient serum.

In this context, nanotechnology has emerged as a promising tool for pathogen detection, providing accurate and sensitive solutions. Gold nanoparticles (GNPs) coated with specific ligands and biomolecules have shown enormous potential for viral [17] and RNA [18] quantification in different samples, including GNPs in solution or immobilized [19]. Bioconjugation of antibodies with GNPs has been explored as an effective alternative to conventional molecular methods, providing a significant improvement in viral detection sensitivity [20,21,22].

Among the properties of GNPs explored for biosensors, localized surface plasmon resonance (LSPR) is noteworthy. LSPR is an optical property characterized by the collective oscillation of conduction electrons on a metal surface when excited by incident light [23]. This oscillation results in a substantial increase in the light absorption and scattering. In rod-shaped GNPs, electron oscillation occurs along the two axes, generating two distinct absorption peaks in the ultraviolet-visible-near-infrared (UV-Vis/NIR) spectrum: one peak corresponding to the transverse plasmon and the other to the longitudinal plasmon [23,24,25].

Longitudinal resonance is affected by the aspect ratio (AR) of GNRs, leading to a shift in the absorption peaks to longer wavelengths when the aspect ratio increases [26,27]. This behavior allows the monitoring of small-molecule adsorption, transforming biological recognition into wavelength-shifting signals in an accurate, sensitive, and real-time manner [28]. Thus, LSPR is a valuable tool for developing biosensors capable of overcoming the limitations of conventional serological and molecular methods and provide superior pathogen detection [17,22,29].

In this study, we developed and validated two LSPR-based GNR biosensors for the rapid and accurate detection of *Orthoflavivirus* in human serum samples and macerated mosquitoes. The proposed biosensors aim to improve early and accurate detection during the acute phase of infection, aiding timely clinical interventions and outbreak management. Additionally, our biosensors aim to address the difficulties in detecting *Orthoflavivirus* in areas where multiple arboviruses are present, providing a quicker, easier, and more effective testing option.

## 2. Materials and Methods

### 2.1. Reagents

Leibovitz’s L-15 medium and fetal bovine serum (FBS) were obtained from Cultilab (Brazil). β-Mercaptoethanol and HiTrap Protein GHP columns were acquired from GE HealthCare Life Sciences (São Paulo, SP, Brazil). The following reagents were purchased from Sigma-Aldrich (St. Louis, MO, USA): RPMI 1640 medium, HEPES buffer (99.5%), cetyltrimethylammonium bromide (CTAB, ≥99%), chloroauric acid (HAuC, ≥99.9%), silver nitrate (AgNO_3_, ≥99%), ascorbic acid (C_6_H_8_O_6_, 99%), N-(3-dimethylaminopropyl)-N′ethylcarbodiimide hydrochloride (EDAC, ≥99.9%), N-hydroxysuccinimide (NHS, 98%), polyethyleneimine (PEI/C_2_H_5_N, *n* = 50% *w*/*v*), and sodium borohydride (NaBH_4_, ≥99%). Hydroquinone was purchased from Vetec.

### 2.2. Human Serum Samples

Human serum samples that tested negative for DENV were obtained from patients treated at the Federal University of Ouro Preto (Brazil) as part of the project titled “Investigation of serological and genetic factors related to the predisposition to the development (predisposition) of severe forms of DENV in Minas Gerais: A study in cities with different epidemiological profiles,” coordinated by Professor Luis Felipe Leomil Coelho at the Federal University of Alfenas. The Research Ethics Committee of the Federal University of Alfenas (Brazil) approved this study, and all patients provided informed consent (Protocol No. 13387313.0.0000.5142). Informed consent was obtained from each participant before sample collection.

### 2.3. Cells and Virus Samples

Hybridomas of 3H5-1 (anti-denv2-ATCC^®^ HB-46TM) and D1-4G2-4-15 (anti-flavivirus-ATCC^®^ HB-112TM) antibodies (Ab) were cultured in RPMI 1640 medium supplemented with 10% FBS, 0.05 mM 2-mercaptoethanol, and 1 mM HEPES and maintained at 37 °C and 5% CO_2_. According to the manufacturer’s instructions, the antibodies were purified using a HiTrap Protein G HP column (Sigma-Aldrich, São Paulo, Brazil).

Samples of DENV 2 (MK506264), collected from New Guinea (Melanesia) in 2007, and ZIKV (PE-243/215), collected from a patient in Pernambuco (Brazil) with mild symptoms, were generously provided by Professor Dr. Erna Kroon from the Microbiology Department at the Federal University of Minas Gerais, Brazil.

*Alphavirus mayaro* (KY618127) sample 20290, initially isolated from *Haemagogus* spp. captured from the Belém-Brasília (Brazil) highway in 1960, was kindly provided by Professor Dr. Mauricio Lacerda Nogueira, Faculty of Medicine of Rio Preto (São Paulo, Brazil).

DENV, ZIKV, and MAYV-Alphavirus were cultured using C6/36 cells in L15 medium supplemented with 10% FBS at 28 °C. After seven days, the cell supernatant was collected by centrifugation at 4000× *g* for 10 min at 4 °C, and the viruses were titrated and expressed as the number of plaque-forming units/mL (PFU/mL). For titration, 6-well plates with 1 × 10^6^ Vero cells/well were infected with each virus diluted in series and incubated for seven days at 37 °C and 5% CO_2_. The titration was performed using 10% formaldehyde and 0.5 mL crystal violet (dilution 1:50 in water).

### 2.4. Mosquito Samples

Non-infected isogenic *Aedes aegypti* mosquitoes were provided by Dr. Paulo Pimenta and Dr. Nagila Secundino from the Research Center René Rachou Fiocruz (Minas Gerais, Brazil). The mosquitoes were fed blood samples contaminated with 10^5^ PFU/mL DENV, ZIKV, or MAYV. For each incubation with GNRs, five mosquitoes were macerated in 200 µL of PBS to obtain a final viral concentration of 2.5 × 10^6^ PFU/mL. A 4× dilution and 6.2 × 10^5^ PFU/mL concentration were used.

### 2.5. Gold Nanorods Synthesis

GNRs were synthesized using seed-mediated synthesis method [30]. Briefly, gold seeds were prepared by mixing 5 mL of HAuCl_4_ (0.05 mM) with 5 mL of CTAB (200 mM) and 1 mM NaBH_4_ under constant stirring. A growth solution was prepared using 0.5 mM HAuCl_4_, 100 mM CTAB, and 4 mM AgNO_3_. Subsequently, 0.1 × 10^3^ mM hydroquinone was added with slow manual stirring, 16 µL of the seed solution was added, and the reaction was maintained at 30 °C for 24 h. Finally, the nanorods were purified by centrifugation at 5600× *g* for 15 min and redispersed in deionized water.

### 2.6. GNRs Characterization

The UV-Vis/NIR absorption spectra were collected using a spectrophotometer UV-1800 (Shimadzu, Kyoto, Japan) in a 10 mm glass cell at 400–1100 nm wavelengths, with 1 nm steps. The data were normalized using PeakFit version 4.12, and GraphPad Prism version 9.0.1 was used to generate the graphs. Nanoparticle stability was analyzed using a Zetasizer (Malvern Panalytical, Malvern, UK). In an aqueous solution, the maximum absorption of surface plasmon resonance (SPR, $\lambda_{max}$) is linearly proportional to the AR (ratio between the nanorod length and diameter) as follows:

$$\lambda_{max}\;=\;95R\;+\;420,$$

where $\lambda_{max}$ is the wavelength at the longitudinal plasmon resonance, given in nanometers, and *R* is the AR [31,32].

Dynamic light scattering (DLS) analysis and zeta potential measurements were performed using a Zetasizer (Nano-Malvern series). For the measurements, 0.01 mL of the sample was added to 1 mL of ultrapure water in a cuvette. At least three readings were obtained for each sample. The analyses were conducted at the Multiuser Laboratory of the Faculty of Pharmacy of the Federal University of Ouro Preto using a Zetasizer (Nano-Malvern series). Transmission electron microscopy (TEM) was performed using a 120 kV Technai Spirit BioTWIN G2-12 microscope (FEI, Hillsboro, OR, USA).

### 2.7. Surface Modifications of GNRs and Bioconjugation with Antibodies

The surfaces of the GNRs were modified with PEI, which mediates nanorod-antibody binding owing to the various reactive amine groups [33]. Then, 100 µL of the GNRs solution was centrifuged at 4000× *g* for 10 min, suspended in 100 µL of PEI solution (0.3% in ultrapure water), and incubated at room temperature (approximately 25 °C) for 30 min in an ultrasonic bath. The solution was centrifuged, suspended in a 0.4 µg/mL antibody solution (3H5 or 4G2) prepared with EDAC/NHS (400 mM:100 mM), and incubated as previously described. GNR-PEI bioconjugated with 3H5 antibodies was named Biosensor-3H5, whereas GNR-PEI bioconjugated with 4G2 antibodies was named Biosensor-4G2.

The surface modification of the GNRs was confirmed using UV-Vis/NIR spectroscopy. Zeta potential measurements were performed to assess the colloidal stability of the nanoparticles and confirm the effectiveness of functionalization with PEI and antibodies.

### 2.8. Biosensor Validation

The biosensors were centrifuged and suspended in 100 µL of PBS containing 10^3^ PFU/mL DENV, ZIKV, or MAYV for validation. MAYV was used as a negative control in all the tests. Diluted human serum (1:3200) and mosquito macerate (1:4) were mixed with different virus concentrations. UV-Vis/NIR absorption spectra were collected after 30 min to determine the biosensor signals.

### 2.9. Methodology Flowchart for Orthoflavivirus Detection Using Functionalized Gold Nanorods

**Figure 1:**
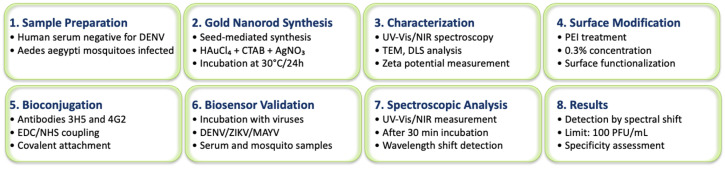
Methodology flowchart for orthoflavivirus detection using functionalized gold nanorods. The eight-step process includes sample preparation, gold nanorod synthesis, characterization, surface modification with PEI, antibody bioconjugation, biosensor validation, spectroscopic analysis, and results interpretation. The method achieves a detection limit of 100 PFU/mL for DENV, ZIKV, and other orthoflaviviruses through localized surface plasmon resonance (LSPR) monitoring.

The eight-step process includes sample preparation, gold nanorod synthesis, characterization, surface modification with PEI, antibody bioconjugation, biosensor validation, spectroscopic analysis, and results interpretation. The method achieves a detection limit of 100 PFU/mL for DENV, ZIKV, and other orthoflaviviruses through localized surface plasmon resonance (LSPR) monitoring (Figure 1).

## 3. Results

**Figure 2:**
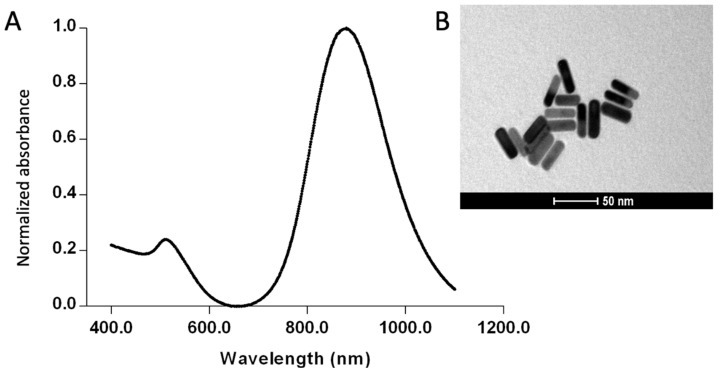
Characterization of the synthesized gold nanorods (GNRs). (**A**) UV-Vis/NIR absorption spectrum, (**B**) transmission electron microscopy image of the GNRs.

In this study, the GNRs synthesized using the seed-mediated method exhibited a transverse plasmon absorption peak at 510 nm and a longitudinal plasmon absorption peak at 927 nm, as shown in the optical absorption spectrum (Figure 2A) From the longitudinal plasmon absorption peak, an aspect ratio of 5.5 was calculated for the GNRs. Rod-like morphology was confirmed by TEM (Figure 2B). DLS analysis revealed an average hydrodynamic diameter of 71.36 ± 6.5 nm, with a polydispersity index (PDI) of 0.144. Zeta potential measurements showed a value of +55.2 ± 3.4 mV. These results indicate a well-defined size of the GNRs, with a homogeneous size distribution and stable dispersion.

**Figure 3:**
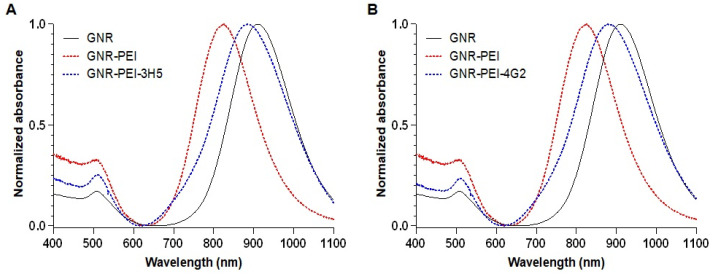
UV-Vis/NIR spectra of the GNR functionalization steps. (**A**) Functionalization with 3H5 antibody. (**B**) Functionalization with 4G2 antibody.

The surfaces of the GNRs were modified with PEI to facilitate antibody bioconjugation. After PEI modification, an 85 nm shift in the longitudinal plasmon peak was observed, indicating successful functionalization of the nanorods. The GNR-PEI was then bioconjugated with antibodies 3H5 (Biosensor-3H5) and 4G2 (Biosensor-4G2), resulting in additional shifts of 59 nm and 54 nm, respectively (Figure 3). The plasmonic shift is the change in the behavior of electron oscillations on the surface of the nanorod, caused by changes in the environment or surface, which manifests as a change in the color of light that the nanorod absorbs or scatters. Zeta potential measurements showed values of +32.3 ± 4.3 mV after PEI functionalization and +28 ± 1.75 mV and +23.8 ± 7.7 mV after bioconjugation with antibodies 3H5 and 4G2, respectively, confirming the effectiveness of antibody binding and the stability of the biosensors.

**Figure 4:**
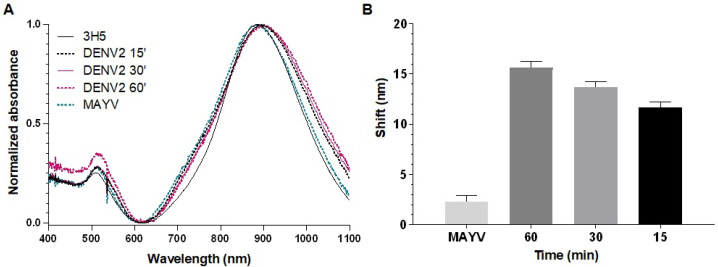
Optical absorption spectra obtained by UV-Vis/NIR spectrophotometry of GNRs conjuged with 3H5 antibody to detect DENV2. (**A**) Biosensor-3H5 (black line) at different reaction times (15 min, dashed black; 30 min, pink; 60 min, dashed pink) after 60 min of incubation with MAYV (dashed green). (**B**) Average longitudinal plasmon absorption shifts after incubation with viruses at different reaction times. Each point represents the mean of triplicates; error bars denote standard deviation.

**Figure 5:**
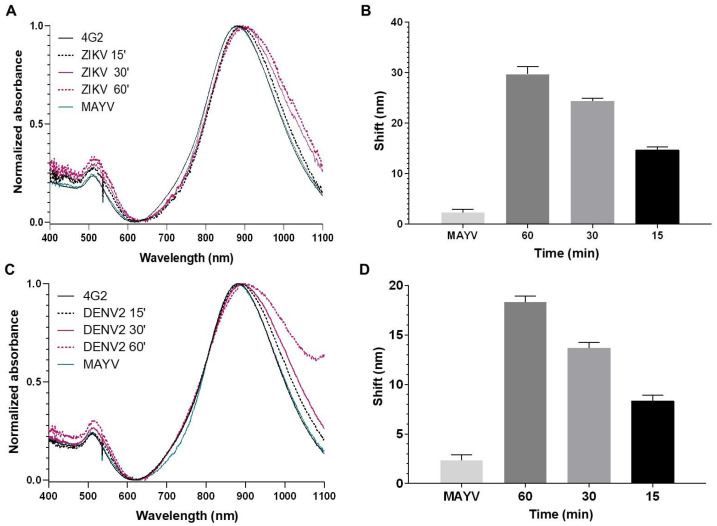
Optical absorption spectra obtained by UV-Vis/NIR spectrophotometry of GNRs conjugated with the 4G2 antibody to detect ZIKV. (**A**,**C**) Biosensor-4G2 (black line) at different reaction times (15 min, dashed black; 30 min, pink; 60 min, dashed pink), with ZIKV or DENV, along with 60 min incubation with MAYV (green). (**B**,**D**) Average longitudinal plasmon absorption shifts after incubation with viruses at different reaction times. Each point represents the mean of triplicates; error bars denote standard deviation.

Initially, we assessed the detection times of the biosensors by incubating them with DENV or ZIKV for 15, 30, and 60 min. After 15 min of incubation at room temperature, virus detection on the biosensors was observed, with a longitudinal plasmonic peak shift of approximately 10 nm (Figure 4 and Figure 5). The specificity of the signal was confirmed because the biosensors could not recognize MAYV (negative control). Biosensor-4G2 showed better sensitivity for ZIKV detection. In this case, a shift of approximately 20 nm was recorded in the first 15 min and up to 30 nm after one hour of incubation.

Next, we aimed to identify the serum dilution that minimized interference from nonspecific binding. To achieve this, the biosensors were tested with serum dilutions of 1:100, 1:200, 1:400, 1:800, 1:1600, and 1:3200 in the presence and absence of 10^3^ PFU/mL of either DENV or ZIKV.

**Figure 6:**
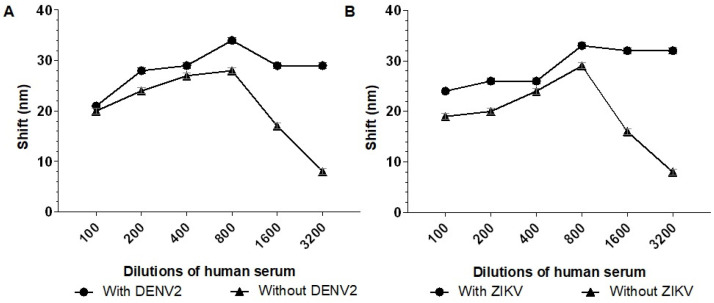
Absorption plasmon spectrum shifts of the biosensors in response to different dilutions of human serum sample. (**A**) Biosensor-3H5 and (**B**) Biosensor-4G2 were incubated with human serum dilutions (1:100, 1:200, 1:400, 1:800, 1:1600, and 1:3200) in the presence of 10^3^ PFU/mL DENV or ZIKV. Each point represents the mean of triplicates; error bars denote standard deviation.

The serum dilution 1:3200 showed better results for both biosensors, with shifts of 21 nm (Biosensor-3H5) and 20 nm (Biosensor-4G2) in the negative serum. Therefore, a 1:3200 serum dilution was chosen to evaluate the detection limit of the biosensor in the presence of different virus concentrations and to evaluate the influence on the specificity of the biosensors when incubated with serum and MAYV (Figure 6).

**Figure 7:**
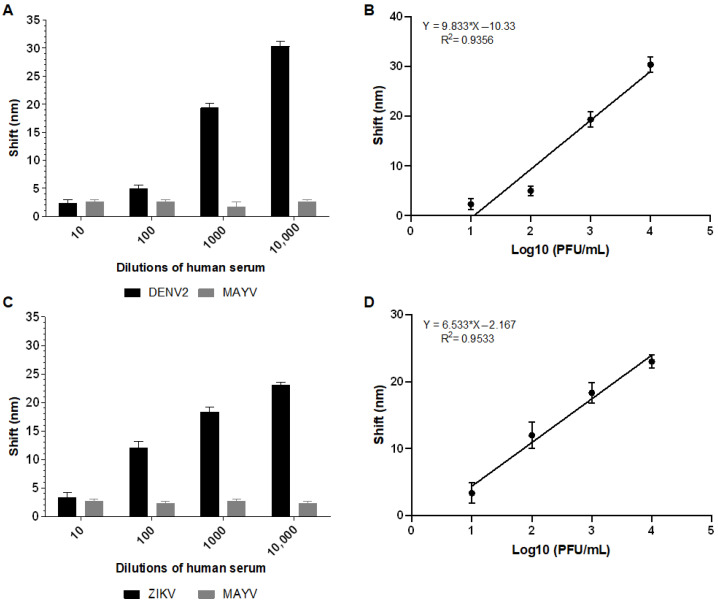
Absorption plasmon spectrum shifts and correlation between viral concentration and biosensor response in human serum sample. (**A**) Plasmon shift detected by Biosensor-3H5 after incubation with DENV or MAYV (negative control). (**B**) Linear regression curve showing the coefficient of determination (R^2^) of Biosensor-3H5, indicating the correlation between the shift and DENV concentration. (**C**) Plasmon shift detected by Biosensor-4G2 after incubation with ZIKV or MAYV (negative control). (**D**) Linear regression curve with the coefficient of determination (R^2^) of Biosensor-4G2, evidencing the correlation between the shift and ZIKV concentration. Each point represents the mean of triplicates; error bars denote standard deviation.

Biosensor-3H5 detected DENV2 at a concentration of 100 PFU/mL in human serum (1:3200) with an average shift of 5 nm (Figure 7A). Similarly, Biosensor-4G2 detected ZIKV in human serum (1:3200) at a concentration of 100 PFU/mL, with an average shift of 12 nm (Figure 7B). At all concentrations and in both biosensors, there was no significant shift for the negative control (MAYV), demonstrating the specificity of the system (Figure 7).

The calibration curve for each biosensor was constructed based on the shift values as a function of the logarithm of the virus concentration in PFU/mL (Log10 PFU/mL). The coefficient of determination (R^2^) value obtained for Biosensor-3H5 was 0.9356 when detecting DENV. The deviation and standard error of the measurements of the lowest concentration tested were calculated from the triplicates, yielding values of 1.15 and 0.66, respectively. For Biosensor-4G2, when detecting ZIKV, the R^2^ was 0.9533, with a deviation and standard error of 1.52 and 0.88, respectively.

In addition to human serum analyses, the performance of the biosensors was evaluated using macerated mosquito samples. For this purpose, 10^3^ PFU/mL of DENV2 or ZIKV (or negative control) was added to different macerated *A. aegypti* mosquito dilutions.

**Figure 8:**
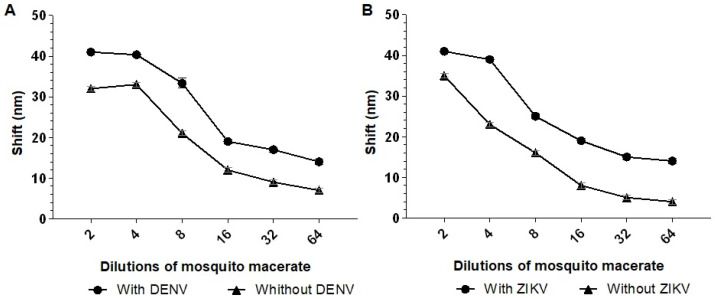
Absorption plasmon spectrum shifts of the biosensors (**A**) Biosensor-3H5 and (**B**) Biosensor-4G2 in different dilutions of mosquito macerate. The biosensors were tested with different dilutions of human serum (1:2, 1:4, 1:8, 1:16, 1:32, and 1:64) with or without the addition of 103 PFU/mL of DENV/ZIKV. Each point represents the mean of triplicates; error bars denote standard deviation.

The best dilution observed to distinguish between the positive and negative controls was 1:4. At a 1:4 dilution, a 9 nm shift in the longitudinal plasmon absorption peak was observed when incubating Biosensor-3H5 with the macerated mosquito sample containing DENV2, compared to the macerated mosquito sample without the virus (Figure 8A). For Biosensor-4G2, the same dilution resulted in a 7 nm shift when incubated with the macerated sample containing ZIKV compared to the sample without the virus (Figure 8B). Based on these results, a 1:4 dilution was chosen for subsequent analysis of the detection limit of the biosensor in macerated mosquito samples.

**Figure 9:**
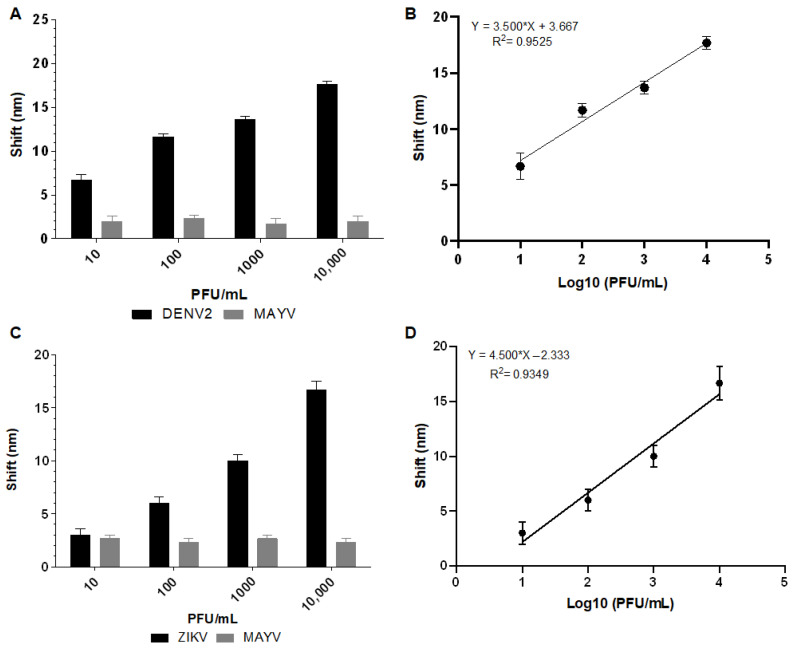
Absorption plasmon spectrum shifts and correlation between viral concentration and biosensor response in macerated mosquito samples. (**A**) Plasmon shift detected by Biosensor-3H5 after incubation with DENV or MAYV (negative control). (**B**) Linear regression curve showing the coefficient of determination (R^2^) of Biosensor-3H5, evidencing the correlation between the shift and DENV concentration. (**C**) Plasmon shift detected by Biosensor-4G2 after incubation with ZIKV or MAYV (negative control). (**D**) Linear regression curve with the coefficient of determination (R^2^) of Biosensor-4G2, evidencing the correlation between the shift and ZIKV concentration. Each point represents the mean of triplicates; error bars denote standard deviation.

The analysis revealed that Biosensor-3H5 could detect DENV at 10 PFU/mL concentrations without significant deviation from the negative control (MAYV). Biosensor-4G2 detected ZIKV at a concentration of 100 PFU/mL (Figure 9). The calibration curve of each biosensor against the macerated mosquito samples was constructed based on the deviation values as a function of the logarithm of the virus concentration in PFU/mL (Log10 PFU/mL). The coefficient of determination (R^2^) obtained for Biosensor-3H5 was 0.9525 for DENV detection. The deviation and standard error of the measurements of the lowest concentration tested were calculated from the triplicates, yielding values of 1.15 and 0.67, respectively. For Biosensor-4G2, in the detection of ZIKV, the R^2^ was 0.9349, with a deviation and standard error of 1 and 0.57, respectively.

These results indicate low variability between the samples and good measurement accuracy. Furthermore, the proposed biosensor can detect *Orthoflavivirus* at low concentrations in human serum and macerated mosquito samples. This consistency in the responses reinforces the potential of biosensors for diagnostic applications in different contexts.

## 4. Discussion

To analyze the performance of the functionalized GNRs for viral particle detection, tests were performed with human serum or with simulations of infected mosquitoes. The GNRs used in this study were synthesized using the seed-mediated method, which employs gold nanospheres as nuclei to promote nucleation and controlled growth, forming rods with well-defined dimensions and morphology. UV-Vis/NIR spectral analysis revealed specific peaks corresponding to the longitudinal and transversal evaluation modes of the GNRs. The intensity of the longitudinal plasmonic detection peak of the GNRs suggests the predominance of nanorods with a low level of spherical particles, as expected [34]. The TEM images confirmed the morphology of the GNRs. DLS and zeta potential analyses showed that the system was monodisperse and stable. These results show that the description is accurate and emphasizes how GNRs can be used to construct biosensors for detecting viral particles.

Significant efforts have been made to develop widely applicable detection platforms that can identify biomolecules with high sensitivity and specificity in diverse clinical samples, such as blood serum, plasma, urine, and cell lysates [35]. Only a small number of studies have shown that GNR-based SPR or LSPR biosensors can capture targets in samples more complicated than water or buffer [36]. The biodegradability and nonspecific binding of biosensors limit their applicability in clinical practice for medical diagnostics [37]. This scenario is especially problematic in diagnosing arboviruses of the *Flaviviridae* family, which often rely on delayed, ineffective, and dependent clinical symptoms [12].

The literature shows that, at peak viremia, DENV1 and DENV2 viruses can present approximately 10^7^ MID50/mL [38], while ZIKV can present approximately 4.8 × 10^7^ GCE/mL (viral genome copy equivalents) [39]. However, after a dilution of 1:3200, these samples will have approximately 3125–15,000 viral particles/mL. The GNR biosensor proposed in this study can detect these viruses at concentrations starting from 100 PFU/mL (Figure 7 and Figure 8), making it a promising detection method.

Approximately 104 PFU/DENV2-midgut were found in mosquitoes after around ten days of feeding on blood, with a decline by the 20th day and a peak of approximately 3.5 × 10^5^ PFU in the head and chest after roughly 30 days of blood feeding [40]. Here, we report the detection of 10 PFU/mL of DENV2 and ZIKV in macerated mosquito samples. Given these results, we believe that the proposed biosensor can directly detect the presence of *Orthoflavivirus* on different days after infection because it can easily detect a concentration approximately 6000 times lower than that currently detected by traditional viral titration methods and real-time PCR. In addition, viral culture and determination of viral particles by electron microscopy are time-consuming, distant from diagnostic routine, and require trained staff.

The sensitivity and specificity of a test depend on the prevalence of the disease, and the results obtained with human serum samples with added viruses do not reflect the real diagnostic value of the test. Therefore, there is still a need to evaluate a larger number of human serum samples to establish the diagnostic performance of the system with the proposed GNRs. In addition, other systems can be tested to ensure efficacy in complex samples, such as coating GNPs with other types of nanoparticles, such as iron oxide nanoparticles and multiwalled carbon nanotubes, which have already demonstrated convincing results for diagnosis and biological detection [41,42].

Preliminary results suggest the great potential of GNRs for the detection of *Orthoflavivirus*, in line with several recent advances in nanotechnology for the diagnosis of arboviruses in mosquitoes [12,43]. The use of GNPs has been explored in several studies, including those of Omar et al. (2018) [44], who detected the DENV protein at a concentration of 39.96 nM^−1^ using SPR with the GNR-IgM complex. Versiani et al. (2020) [36] developed a biosensor based on GNRs to detect anti-DENV monoclonal antibodies. Based on these results, we believe that the biosensors proposed in this study are highly sensitive and specific for detection, even in complex samples such as human serum, providing a direct and more effective option compared to the methods currently used in clinical and epidemiological practices.

In localized surface plasmon resonance (LSPR) assays using gold nanorods functionalized with monoclonal antibodies for the recognition of viral particles, matrix effects and sample-to-sample variability are important factors to consider. In diluted human serum, the presence of proteins, lipids, and salts may interfere with antigen–antibody interactions or cause nonspecific adsorption to the sensor surface, potentially affecting signal stability and sensitivity. Similarly, mosquito macerates contain complex biological material, including proteases, nucleases, and cellular debris, which may impact nanoparticle behavior or obstruct binding sites. Despite these challenges, the biosensors demonstrated consistent performance across independent experiments, suggesting that the functionalization strategy and sample preparation protocols were effective in minimizing such effects. Nevertheless, some degree of signal variability was observed and is likely attributable to inherent differences in the biological composition of each sample type.

However, the inclusion of a preliminary step involving the capture of viruses using magnetic particles conjugated to antibodies can improve the detection capacity in complex samples, especially through LSPR, since the turbidity of the medium hinders the acquisition of reliable results in these assays. It will also be necessary to compare the presented results with other similar studies reported in the literature using real serum and mosquito macerate samples containing unknown concentrations of viral particles, in order to unequivocally establish the advantages and limitations of this method. The advantages and disadvantages between traditional methods and Gold Nanorod Plasmon Resonance are listed in the Table 1.

## 5. Conclusions

In this study, LSPR-based GNRs biosensors were developed to detect *Orthoflavivirus*. Biosensor-3H5 detected DENV2 at levels as low as 10 PFU/mL, whereas Biosensor-4G2 successfully detected ZIKV at levels as low as 100 PFU/mL in human serum and mosquito macerates. The proposed biosensor offers a promising alternative for the direct detection of *Orthoflavivirus*. Further studies are warranted to determine the minimum PFU required to detect other viruses such as *Orthoflavivirus nilense*, *Orthoflavivirus flavi*, and other DENV serotypes. The proposed biosensors accurately and sensitively identified the target virus in both pure and more complex samples, such as human blood and ground-up mosquito samples. Their rapid detection capability, combined with the potential for large-scale production, suggests that these biosensors could significantly reduce the costs of direct diagnosis of viral diseases with similar clinical presentations and be useful in virological surveillance. Thus, the presented data emphasize the possible use of the proposed biosensors as a novel and practical approach for the direct diagnosis of *Orthoflavivirus*.

## Figures and Tables

**Table 1:** Comparative performance characteristics of traditional methods (PCR/serology) and Gold Nanorod Localized Surface Plasmon Resonance (GNR-LSPR) biosensors across ten key operational parameters relevant to clinical and field diagnostic applications.

Feature	Traditional Methods (PCR/Serology)	Gold Nanorod Plasmon Resonance (GNR-LSPR)	References
Sensitivity	High (PCR), Moderate (Serology)	High (especially with specific functionalized antibodies)	[25,45,46]
Specificity	High (PCR), Variable (Serology, prone to cross-reactivity)	Depends on antibody specificity; high when using well-characterized antibodies	[25,45,47,48]
Time to Result	Several hours to days	Rapid (typically minutes to a few hours)	[49,50,51]
Infrastructure Requirement	Requires advanced lab equipment and trained personnel	Minimal equipment; potentially portable systems	[52,53,54]
Cost per Test	High (especially PCR)	Moderate to low (after nanoparticle preparation)	[55,56]
Multiplexing Capability	Limited (PCR can multiplex with design; serology is usually single target)	Limited, but can be enhanced with sensor array design	[57,58,59]
Cross-reactivity Risk	High for serology (especially in co-endemic regions)	Low if antibody selection is optimal	[47,48,60]
Reusability of Components	Generally single-use	Sensors may be reusable depending on design	[61,62]
Field Applicability	Limited	High potential for point-of-care or field use	[63,64]
Detection in Mosquito Samples	Possible but labor-intensive (RNA extraction + PCR)	Direct detection possible with simple sample preparation	[65,66,67]

## Data Availability

All relevant analyses derived from the data are presented in this manuscript. Further inquiries should be directed to the corresponding author.

## Abbreviations

The following abbreviations are used in this manuscript:

DENV	*Orthoflavivirus dengue*
ZIKV	*Orthoflavivirus zikaense*
GNPs	Gold Nanoparticles
GNRs	Gold Nanorods
LSPR	Localized Surface Plasmon Resonance
AR	Aspect Ratio
FBS	Fetal Bovine Serum
PEI	Polyethyleneimine
CTAB	Cetyltrimethylammonium Bromide
HAuCl_4_	Chloroauric acid
AgNO_3_	Silver nitrate
EDAC	N-(3-dimethylaminopropyl)-N′ethylcarbodiimide hydrochloride
NHS	N-hydroxysuccinimide
NaBH_4_	Sodium borohydride
MAYV	*Alphavirus mayaro*
PDI	Polydispersity index
DLS	Dynamic Light Scattering
TEM	Transmission Electron Microscopy
SPR	Surface Plasmon Resonance
PFU	Plaque Forming Units
